# Supplementary material for: MCNMF-Unet: a mixture Conv-MLP network with multi-scale features fusion Unet for medical image segmentation
Source: PeerJ Comput Sci. 2024 Jan 12;10:e1798. doi: 10.7717/peerj-cs.1798 (PMC10803052; doi:10.7717/peerj-cs.1798)
Supplement: Supplemental Information 1 [file peerj-cs-10-1798-s001.docx]

**Layer Normalization vs. Batch Normalization in the MCG Module: Rationale and Results**

In the MLP Cross Gating (MCG) module, we have opted to use Layer Normalization (LN) layers instead of Batch Normalization (BN). This decision stems from the rationale of normalizing the activations within each layer independently. LN has been demonstrated to enhance the generalization and stability of deep neural networks in computer vision tasks, mitigating the impact of covariate shift within each layer and facilitating more effective learning.

While LN is typically associated with Natural Language Processing (NLP) tasks, its application in computer vision has gained prominence with the emergence of Vision Transformers and related works. Vision Transformers have effectively employed LN to normalize activations within each transformer block, contributing to their strong performance in image recognition tasks.

Comparatively, BN is a widely-used normalization technique in computer vision, normalizing activations across the entire batch and proving effective in improving training and generalization of deep neural networks. However, in the context of the MCG module, the choice of LN over BN may be justified by its capacity to independently normalize activations within each layer, potentially offering better control over the normalization process.

In preliminary experiments, we replaced the LN layer in the MCG module with a BN layer. As shown in Table 1, we conducted comparative experiments on the BUSI, ISIC2018, and CVC-ClinicDB datasets, and the results further supported the suitability of LN for the MCG module. LN's independent normalization may help to better preserve local and fine features extracted by the convolution operation while extending the feature map to a global receptive field.

**Table 1：**Comparison of Layer Normalization (LN) and Batch Normalization (BN) in the MLP Cross Gating (MCG) Module

| Variant | BUSI | | ISIC2018 | | CVC-ClinicDB | |
| --- | --- | --- | --- | --- | --- | --- |
|  | IoU(%) | F1_score(%) | IoU(%) | F1_score(%) | IoU(%) | F1_score(%) |
| BN | 73.80 | 84.76 | 79.81 | 87.95 | 83.91 | 91.16 |
| LN | 74.19 | 84.89 | 81.99 | 89.96 | 84.04 | 91.18 |
